# Serum Leptin Is a Biomarker of Malnutrition in Decompensated Cirrhosis

**DOI:** 10.1371/journal.pone.0159142

**Published:** 2016-09-01

**Authors:** Vikrant Rachakonda, Amir A. Borhani, Michael A. Dunn, Margaret Andrzejewski, Kelly Martin, Jaideep Behari

**Affiliations:** 1 Department of Medicine, Divisions of Gastroenterology, Hepatology, and Nutrition, University of Pittsburgh, Pittsburgh PA 15213, United States of America; 2 Department of Radiology, University of Pittsburgh School of Medicine, Pittsburgh, PA, 15213, United States of America; Taipei Veterans General Hospital, TAIWAN

## Abstract

**Background and Aims:**

Malnutrition is a leading cause of morbidity and mortality in cirrhosis. There is no consensus as to the optimal approach for identifying malnutrition in end-stage liver disease. The aim of this study was to measure biochemical, serologic, hormonal, radiographic, and anthropometric features in a cohort of hospitalized cirrhotic patients to characterize biomarkers for identification of malnutrition.

**Design:**

In this prospective observational cohort study, 52 hospitalized cirrhotic patients were classified as malnourished (42.3%) or nourished (57.7%) based on mid-arm muscle circumference < 23 cm and dominant handgrip strength < 30 kg. Anthropometric measurements were obtained. Appetite was assessed using the Simplified Nutrition Appetite Questionnaire (SNAQ) score. Fasting levels of serum adipokines, cytokines, and hormones were determined using Luminex assays. Logistic regression analysis was used to determine features independently associated with malnutrition.

**Results:**

Subjects with and without malnutrition differed in several key features of metabolic phenotype including wet and dry BMI, skeletal muscle index, visceral fat index and HOMA-IR. Serum leptin levels were lower and INR was higher in malnourished subjects. Serum leptin was significantly correlated with HOMA-IR, wet and dry BMI, mid-arm muscle circumference, skeletal muscle index, and visceral fat index. Logistic regression analysis revealed that INR and log-transformed leptin were independently associated with malnutrition.

**Conclusions:**

Low serum leptin and elevated INR are associated with malnutrition in hospitalized patients with end-stage liver disease.

## Introduction

Malnutrition consists of skeletal muscle loss with or without loss of adipose tissue mass. It is frequently observed in cirrhosis with a reported prevalence as high as 65–90% [1–3]. Multiple mechanisms contribute to the development of malnutrition in end-stage liver disease, including decreased oral intake, altered energy substrate utilization, and hypercatabolism [3]. The clinical significance of malnutrition in liver disease is widely recognized, as nutritional status was a component of the original Child-Turcott score [4]. Furthermore, multiple studies have identified malnutrition as an independent predictor of mortality [5, 6], hepatic decompensation [7, 8] (ascites, hepatic encephalopathy, spontaneous bacterial peritonitis, variceal bleeding), and quality of life [9].

Despite its prognostic significance in cirrhosis, malnutrition remains underdiagnosed in clinical practice [10]. Although multiple noninvasive methods for assessing nutritional status have been studied, there is no consensus as to the optimal approach for identifying malnutrition in end-stage liver disease. Anthropometric measurements including body mass index and waist circumference are difficult to interpret in cirrhotic patients due to the presence of ascites and peripheral edema [3]. Serum levels of proteins and catabolic markers may be reduced in cirrhotic patients due to impaired hepatic synthetic function and often do not reflect nutritional stores [11, 12]. Some advances, however, have been made. In a study of cirrhotic patients awaiting liver transplantation, the combination of mid-arm muscle circumference < 23 cm and hand grip strength < 30 kg had a 94% sensitivity and 97% negative predictive value when compared to body cell mass as determined by isotope dilution technique [13, 14]. Cross-sectional measurement of paraspinal skeletal muscle area using computed tomography has been evaluated in both pre- and post-transplant settings [15–17]. Nonetheless, there are no global markers of malnutrition that capture the multifactorial pathogenesis of this condition in cirrhosis.

Although malnutrition adversely impacts clinical outcomes in cirrhosis, early identification of malnutrition in cirrhotic patients remains challenging. Therefore, the goal of this study was to measure biochemical, serologic, hormonal, radiographic, and anthropometric features in a cohort of hospitalized cirrhotic patients to uncover objective biomarkers for identification and monitoring of malnutrition.

## Methods

### Study Design and Participants

This prospective, observational cohort study was approved by the institutional review board (IRB) at the University of Pittsburgh. Fifty-two cirrhotic patients were enrolled at the University of Pittsburgh Medical Center Presbyterian Hospital in Pittsburgh, Pennsylvania from November 2013 to June 2014. Informed consent was obtained prior to study inclusion from either the subject or a designated representative. Inclusion criteria included age over 18 years, ability and willingness to provide informed consent, and ability to perform diagnostic tests. Exclusion criteria included any history of solid organ transplantation or hematopoietic stem cell transplant, death or transplantation during the index hospitalization, and discharge with hospice care.

### Clinical Evaluation

Participants underwent a structured clinical assessment within 48 hours prior to hospital discharge. Age, gender, self-reported ethnicity, and past medical history were obtained from the medical record and through subject interviews. Records were reviewed by two hepatologists (J.B, V.R.) to establish the etiology of cirrhosis, hospital admission diagnoses, cirrhosis-related complications, including varices, hepatic encephalopathy, hepatic hydrothorax, and hepatocellular carcinoma, and to record prior transjugular intrahepatic portosystemic shunts (TIPS) procedures. On physical examination, the presence of jaundice was documented, and ascites was graded as absent (grade = 0), visible only by ultrasound (grade 1), visible ascites with bulging flanks (grade = 2), and tense ascites (grade = 3) [18].

All laboratory tests were obtained after an overnight fast. Routine clinical laboratory measurements were performed at the University of Pittsburgh Clinical Laboratories. These included serum glucose, sodium, potassium, chloride, bicarbonate, urea nitrogen, calcium, creatinine, alanine aminotransferase (ALT), aspartate aminotransferase (AST), total bilirubin, alkaline phosphatase, total protein, albumin, complete blood cell counts, and international normalized ratio (INR). The MELD score was calculated as follows [19]:

$$\text{MELD }=\;\left(0.957\;\times \text{ log }\left[\text{creatinine }\left(\frac{\text{mg}}{\text{dl}}\right)\right]+\;0.378\;\times \text{ log }\left[\text{total bilirubin }\left(\frac{\text{mg}}{\text{dl}}\right)\right]+\;1.120\;\times \text{ log }\left(\text{INR}\right)+\;0.643\right)\text{ X }10$$

Patients who received at least two renal replacement treatments within one week of enrollment were assigned a creatinine value of 4.0 mg/dl. Child-Turcott-Pugh Score and Class were determined as previously described [20].

### Nutritional Evaluation

Subjects were measured on a standard upright weight scale, and this weight was designated as wet weight. Dry weight was determined as wet weight with 5% subtracted if grade 1 ascites was present, 10% subtracted if grade 2 ascites was present, and 15% subtracted if grade 3 ascites is present. If a patient underwent cumulative paracentesis of over 10 liters during the hospitalization, the post paracentesis weight was considered the dry weight. An additional 5% was subtracted if lower extremity pitting edema was present [16]. Height was measured twice using a standard ruler, and the average of two readings was recorded. Wet/dry body mass index (BMI) were calculated from the wet/dry weights and height as follows:

$$\text{BMI}\;=\;\text{weight }\left(\text{kg}\right)/\text{height }{\left(\text{m}\right)}^{2}$$

Dominant arm mid arm circumference was assessed with a flexible measuring tape at the midpoint between the acromion and epicondylar process. Triceps skinfold thickness was measured at the same location using the Lange Skinfold Caliper (Cambridge Scientific Industries, Cambridge, MD, USA). Mid-arm muscle circumference was calculated as follows [21]:

Mid−arm muscle circumference = Mid−arm circumference (cm)−0.314 × Triceps skinfold thickness (mm)

Handgrip strength was assessed using the Jamar dynamometer (J.A. Preston Co., Jackson, MI, USA). Patients underwent three consecutive measurements of grip strength in the dominant hand, and the mean grip strength was then calculated. A patient was classified as malnourished if both mid arm muscle circumference was < 23 cm and handgrip strength was < 30 kg [13, 14].

Subjects were asked to walk 15 feet at their usual pace, and completion times were measured with a stopwatch. The mean walking time of three attempts was calculated. Patients who were unable to walk were assigned the longest measured walking time in the cohort. Finally, appetite was assessed using the Simplified Nutritional Appetite Questionnaire (SNAQ) score [22]; anorexia was defined as a SNAQ score ≤ 14.

### Radiographic Analysis of Body Composition

Thirty-eight patients (73.1%) underwent abdominal axial computed tomography (CT), and seven patients (13.4%) underwent abdominal axial magnetic resonance imaging (MRI) within three months of study enrollment. Images were analyzed with SliceOmatic (Version 4.3) software (Tomovision, Montreal, Quebec, Canada) to segment paraspinal skeletal muscle and visceral adipose tissue using previously validated Hounsfield Unit (HU) cutoffs [16, 23]. Two consecutive axial CT images at the level of the L3 vertebral body and immediately below toward the iliac crest were chosen for analysis in each patient. Axial CT images were reconstructed at 5 mm thickness and with no inter-slice gap; the mean skeletal muscle and visceral fat areas (in cm^2^) derived from the two images was reported. Liver protocol MRI does not routinely extend below the L2/L3 level. Therefore, the single most caudal image from non-fat-saturated spoiled echo T1-weighted sequences was chosen for analysis [24]. The most cranial level included as at the level of the L1/L2 intravertebal disk. The MRI images were acquired at 8 mm thickness with 2 mm inter-slice gap. MRI examinations were performed using 1.5 Tesla (n = 6) and 3 Tesla (n = 2) magnets. The skeletal muscle index and visceral fat index were calculated as follows [15, 25]

$$\text{Skeletal muscle index}\;=\;\text{skeletal muscle area }\left({\text{cm}}^{2}\right)/\text{ height }\left({\text{cm}}^{2}\right)$$

$$\text{Visceral fat index}=\text{visceral m area }\left({\text{cm}}^{2}\right)/\text{ height }\left({\text{cm}}^{2}\right)$$

### Measurement of Serum Cytokines, Adipokines and Hormones

Twenty milliliters of blood were collected in sterile EDTA-containing tubes for serologic analysis. Samples were obtained between 4 AM and 6 AM. Serum was separated by centrifugation and stored at − 80°C until testing. Levels of cytokines (TNF-α, IL-6, IL-1β, IL-8, GM-CSF), adipokines (adiponectin, leptin, resistin, and PAI-1), and peptide hormones (insulin, ghrelin, gastric inhibitory peptide (GIP), glucagon-like peptide 1 (GLP-1), and amylin) were measured on a Luminex platform at the University of Pittsburgh Genomics and Proteomics Core Facility (Pittsburgh, PA).

### Statistical Analysis

Statistical analysis was performed with Stata Version 11 (StataCorp, College Station, TX) and GraphPad version 6.0 (GraphPad Software, La Jolla, CA). Clinical and demographic characteristics were reported with absolute frequencies, percentages, medians, and interquartile ranges (IQR). Comparisons between groups were performed using Fischer’s exact test for categorical variables, while the Mann-Whitney *U* test was used for continuous variables. Correlation between variables was determined using Spearman’s rank correlation coefficient (ρ). Levels of cytokines, adipokines, and hormones were assessed for normal distribution using the Shapiro-Wilk test, and log-transformation was performed on non-normal variables for imputation into regression analyses. Logistic regression was used to determine features associated with malnutrition, and variables with *p* < 0.1 in univariate models were included in multivariate analysis. To generate a parsimonious model, a backward stepwise elimination algorithm was performed to determine regressors for the final model. Variance inflation factors were assessed in the final model to ensure no collinearity between explanatory variables. The prognostic accuracy of the multivariate model was evaluated with area under receiver-operator curve (AUROC) analysis. Two-sided *p* values < 0.05 were considered statistically significant.

## Results

### Demographic, clinical, and nutritional characteristics of patients

Clinical and demographic characteristics are summarized in Table 1. Twenty-two patients (42.3%) met functional and anthropometric criteria for malnutrition. The median age was 57 years (IQR 50.5–62.5 year), and 42.3% were female. The majority of patients were white, and the leading etiology of cirrhosis was alcoholic liver disease. Eight subjects had previous TIPS placement (15.4%), and five had hepatocellular carcinoma (9.6%). 65.4% of subjects had Child’s Class C cirrhosis, and the median MELD score was 18 (IQR 16–22). As summarized in S1 Table, hospitalization diagnoses were similar between patients with and without malnutrition, and length of hospitalization was similar between nourished and malnourished subjects [6.0 (3.0–10.0) vs. 5.5 (4.0–9.0) days, *p* = 0.997]. There were no significant differences in serum sodium, albumin, bilirubin, and creatinine levels in patients with and without malnutrition. Malnourished participants had higher MELD scores [20 (18–24) vs. 17 (14–20), *p* = 0.007], and this was due to higher INR [1.7 (1.5–2.0) vs. 2.1 (2.0–5.0), *p* = 0.01].

**Table 1:** Clinical and Demographic Characteristics of Patients.

Characteristics	Total	Malnourished	No malnutrition	*P*
	(N = 52)	(N = 22)	(N = 30)	
**Age, yrs**	57.5 (50.5–62.5)	57.0 (48.8–62.0)	58.0 (51.0–63.5)	0.753
**Female, n (%)**	22 (42.3%)	11 (50%)	11 (36.7%)	0.249
**Ethnicity, n (%)**	***	***	***	0.672
** Caucasian**	50 (96.2%)	21 (95.5%)	29 (96.7%)	
** Black**	2 (3.8%)	1 (4.5%)	1 (3.3%)	
**Etiology of Cirrhosis, n (%)**	***	***	***	
** Hepatitis C**	15 (28.8%)	6 (27.3%)	9 (30.0%)	0.541
** Hepatitis B**	2 (3.8%)	1 (45.5%)	1 (3.3%)	0.672
** Ethanol**	24 (46.2%)	8 (36.4%)	16 (53.3%)	0.176
** NAFLD/cryptogenic**	17 (32.7%)	8 (36.4%)	9 (30.0%)	0.425
** Primary Sclerosing Cholangitis**	2 (3.8%)	2 (9.1%)	0 (0%)	0.174
** Other**	1 (1.9%)	1 (4.5%)	0 (0%)	0.423
**Cirrhosis-Related Complications (n,%)**	***	***	***	
** Esophageal Varices**	41 (78.8%)	20 (90.1%)	21 (70.0%)	0.067
** Ascites and/or hydrothorax**	50 (96.2%)	21 (95.5%)	29 (96.7%)	0.381
** Hepatic Encephalopathy**	41 (78.8%)	17 (72.3%)	24 (80.0%)	0.538
**Child-Turcott-Pugh Score (n, %)**	***	***	***	0.606
** Class A**	2 (3.8%)	1 (4.5%)	1 (3.3%)	
** Class B**	16 (30.8%)	5 (22.7%)	11 (36.7%)	
** Class C**	34 (65.4%)	16 (72.7%)	18 (60%)	
**MELD**	18 (16–22)	20 (18–24)	17 (14–20)	0.007
**Sodium (mmol/L)**	135 (131–136)	135 (133–136)	134 (130–137)	0.334
**Albumin (g/L)**	3.0 (2.6–3.4)	3.1 (2.8–3.5)	3.0 (2.5–3.4)	0.423
**Bilirubin (mg/dl)**	2.6 (1.7–4.3)	2.7 (2.0–5.0)	2.3 (1.6–4.2)	0.239
**INR**	1.8 (1.6–2.1)	2.1 (1.7–2.4)	1.7 (1.5–2.0)	0.01
**Creatinine (mg/dl)**	0.8 (0.9–1.3)	1.0 (0.7–1.6)	0.9 (0.8–1.1)	0.495
**WBC count (x10**^**3**^**/ml)**	4.5 (3.4–7.2)	4.1 (3.2–6.1)	4.7 (3.8–7.7)	0.204
**Hemoglobin (gm/dl)**	9.2 (8.1–11.3)	8.8 (7.9–9.5)	10.3 (8.3–11.8)	0.068
**Platelet count (x10**^**3**^**/ml)**	70 (53–104)	67 (56–95)	70 (53–106)	0.76
**TIPS placement (n, %)**	8 (15.4%)	2 (9.1%)	6 (20.0%)	0.25
**Hepatocellular Carcinoma (n, %)**	5 (9.6%)	2 (9.1%)	3 (10.0%)	0.648

Clinical and demographic characteristics of patients stratified by nutritional status. All values are expressed as medians and IQRs unless otherwise specified. Continuous variables were compared with Mann-Whitney *U* test. Categorical variables were compared with Fisher's exact test. INR, international normalized ratio; MELD, Model for End-stage Liver Disease; TIPS, transjugular intrahepatic portosystemic shunt; WBC, white blood cell.

Anthropometric, functional, and nutritional features are depicted in Table 2. Patients without malnutrition had higher wet and dry BMI, mid arm circumference, triceps skinfold thickness, mid arm muscle circumference, and handgrip strength compared to malnourished subjects. In a subset of 45 patients with cross-sectional abdominal imaging within three months of study enrollment, both skeletal muscle index [39.62 (IQR 34.08–44.44) cm^2^/m^2^ vs. 46.64(43.25–58.84) cm^2^/m^2,^ *p* = 0.005] and visceral fat index [18.29 (12.21–34.46) cm^2^/m^2^ vs. 52.40 (32.55–81.61) cm^2^/m^2^, *p* = 0.003] were significantly lower in malnourished patients. Visceral fat index was significantly correlated with triceps skinfold thickness, wet BMI, and dry BMI; skeletal muscle index was significantly correlated with visceral fat index, mid arm muscle circumference, wet BMI, and dry BMI (S1 Fig). There were no differences in SNAQ scores, frequency of anorexia, weight loss, or 15 foot walking time between nourished and malnourished patients.

**Table 2:** Anthropometric, Functional, and Nutritional Features of Patients.

Characteristics	Total	Malnourished	No malnutrition	*P*
	(N = 52)	(N = 22)	(N = 30)	
**Wet weight (kg)**	88.4 (75.2–103.5)	76.8 (69.0–85.8)	99.4 (84.6–113.0)	< 0.001
**Dry weight (kg)**	81.0 (66.5–91.5)	66.6 (58.6–81.7)	83.2 (78.0–103.1)	< 0.001
**Wet BMI (kg/m**^**2**^**)**	30.8 (26.0–35.5)	26.2 (24.3–30.8)	34.8 (28.1–38.6)	0.001
**Dry BMI (kg/m**^**2**^**)**	26.3 (22.8–31.9)	22.8 (20.5–26.3)	29.6 (25.3–34.3)	< 0.001
**Mid-arm circumference (cm)**	28.0 (25.8–31.2)	25.0 (23.0–27.0)	30.5 (28.0–33.0)	< 0.001
**Triceps skin fold thickness (mm)**	14.2 (5.6–19.8)	12.8 (4.0–17.8)	14.3 (8.3–21.9)	0.069
**Mid-arm muscle circumference (cm)**	23.7 (22.0–26.6)	21.4 (20.3–22.3)	26.2 (24.7–27.3)	< 0.001
**Hand grip strength (kg)**	18.3 (16.3–26.2)	17.5 (16.1–19.6)	23.3 (16.8–30.3)	0.027
**SNAQ Questionnaire (Score)**	13 (11–15)	13 (11–15)	13 (11–16)	0.794
**Anorexia (n, %)**	35 (67.3%)	15 (68.2%)	20 (66.7%)	0.575
**Weight loss ≥ 10 pounds in last year (n, %)**	34 (65.4%)	17 (77.3%)	17 (56.7%)	0.105
**15 foot walking time (seconds)**	7.8 (5.9–15.3)	7.6 (6.1–9.9)	9.2 (5.9–16.4)	0.496

Anthropometric, appetite, and functional features of patients stratified by nutritional status. All values are expressed as medians and IQR unless otherwise specified. Continuous variables were compared with Mann-Whitney *U* test. Categorical variables were compared with Fisher's exact test. SNAQ, Simplified Nutritional Appetite Questionnaire.

### Serum leptin levels are decreased in malnourished cirrhotic patients

**Fig 1:**
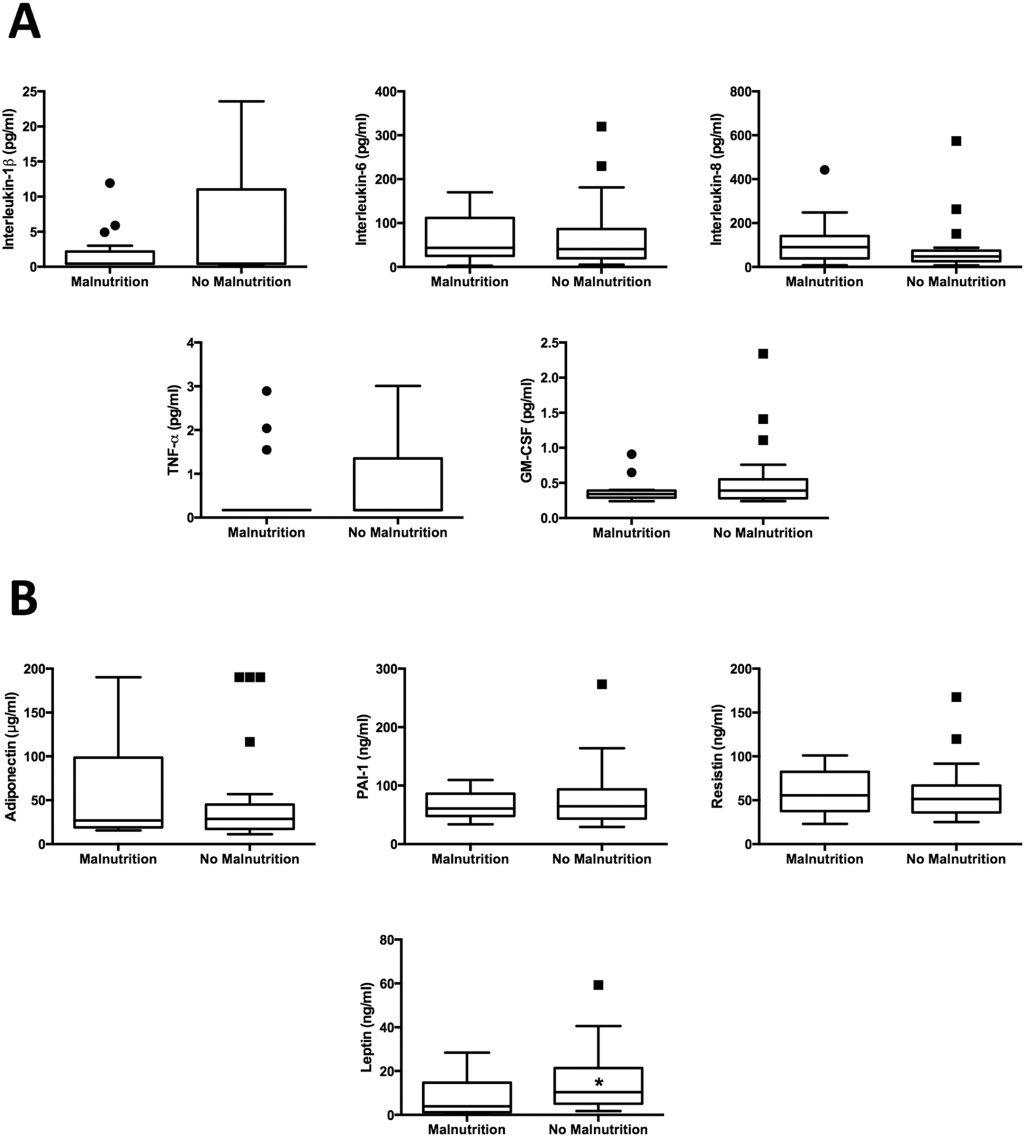
Tukey box whisker plots of circulating concentrations of (A) inflammatory cytokines [interleukin-1β, interleukin-6, interleukin 8, tumor necrosis factor-α (TNF-α) and granulocyte-macrophage colony stimulating factor (GM-CSF)] and (B) adipokines [adiponectin, plasminogen activator inhitior-1 (PAI-1), resistin, and leptin] in cirrhotic patients with and without malnutrition. *, *p* < 0.05.

**Fig 2:**
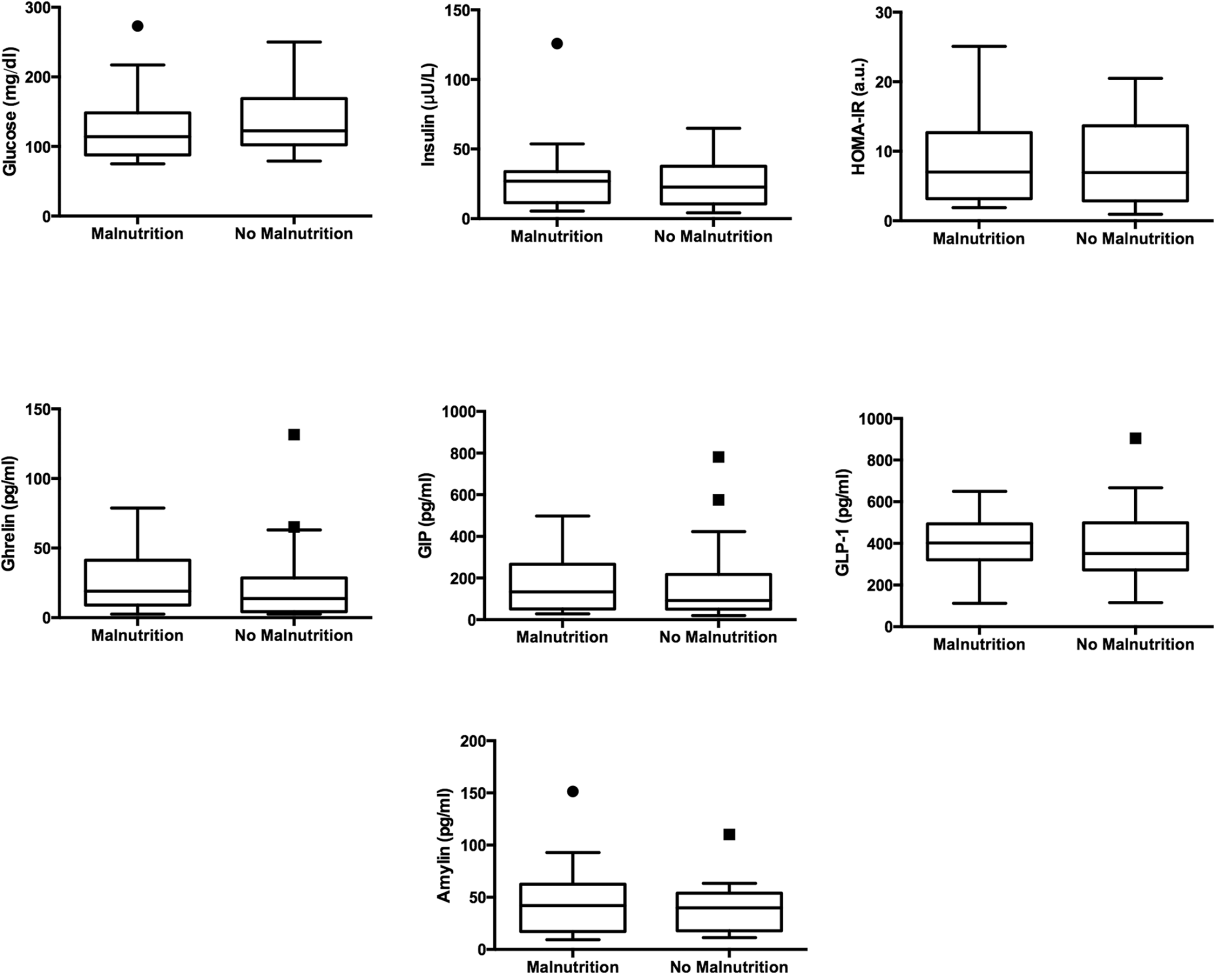
Tukey box whisker plots of circulating concentrations of insulin resistance markers and metabolic hormones. GIP-1, gastric inhibitory peptide; GLP-1, glucagon-like peptide 1; HOMA-IR, homeostatic model assessment insulin resistance; *, *p* < 0.05.

We also measured inflammatory cytokines, adipokines, appetite regulatory hormones, and insulin resistance (Figs 1 and 2; S2 Table). Levels of inflammatory cytokines were not elevated in patients with malnutrition, despite higher MELD scores. There were no significant differences in insulin resistance, and most appetite regulation hormones were similar between groups. Malnourished patients had decreased triceps skinfold thickness, a surrogate marker of fat mass. Among measured adipokines, serum leptin [3.88 (1.52 − 11.02) ng/ml vs. 10.39 (5.46 − 21.06) ng/ml, *p* = 0.011] was significantly lower in malnourished patients.

**Fig 3:**
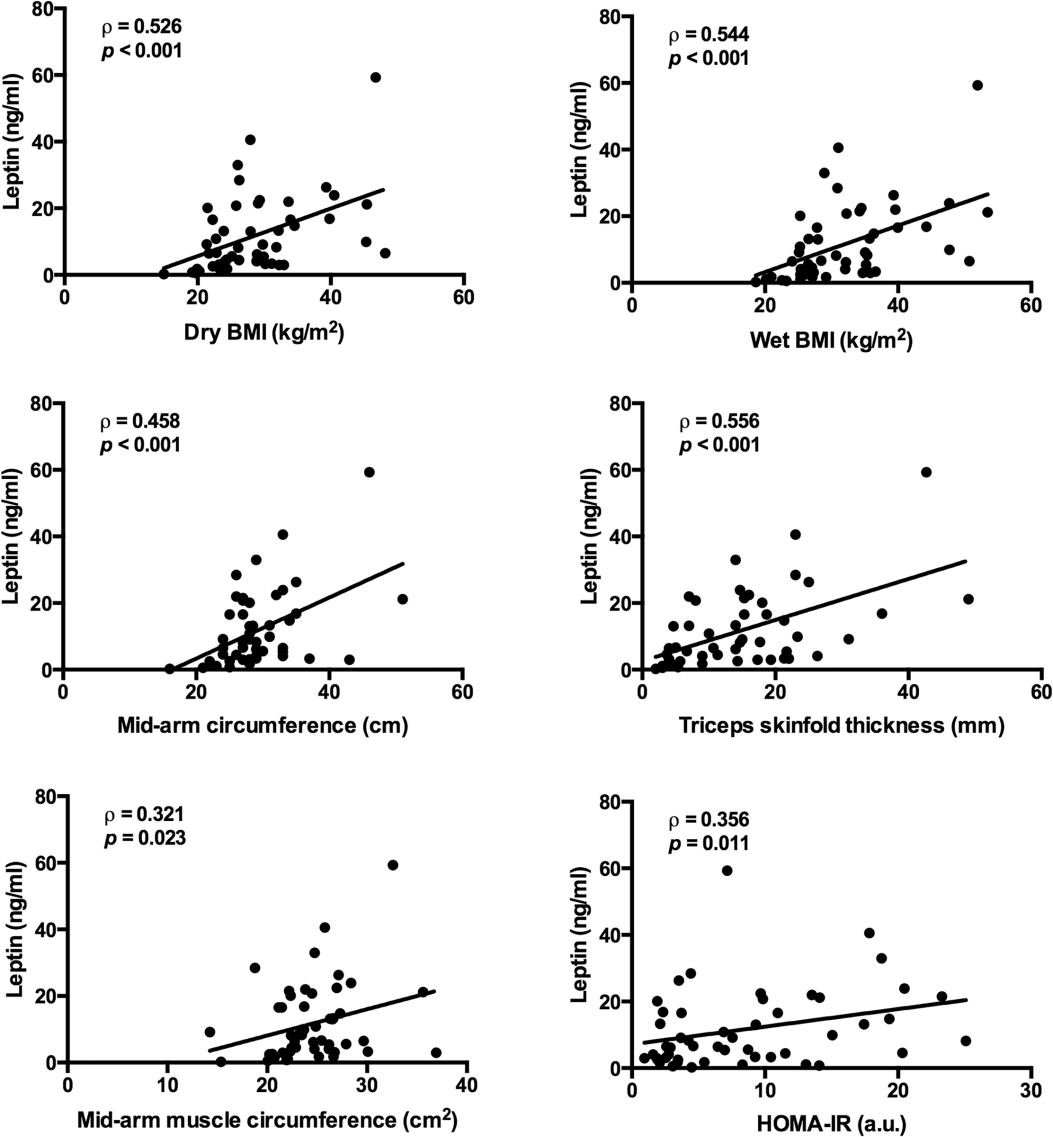
Spearman correlations of serum leptin with dry BMI, wet BMI, mid-arm circumference, triceps skinfold thickness, mid-arm muscle circumference, and HOMA-IR.

As serum leptin levels were reduced in malnourished patients, we assessed correlations between leptin and patient characteristics using Spearman rank coefficients (Fig 3; S3 Table). Among anthropometric features, wet and dry BMI, mid arm circumference, triceps skinfold thickness, and mid arm muscle circumference were positively correlated with leptin levels. In the subset of patients with available cross-sectional imaging analysis, leptin levels were positively correlated with both visceral fat and skeletal muscle indices (S2 Fig).

Among metabolic markers, only HOMA-IR exhibited significant, positive correlation with leptin. On the other hand, MELD, clinical chemistries, inflammatory cytokines and other adipokines were not correlated. There were no significant associations with markers of appetite or with appetite-regulatory hormones. Finally, leptin levels were not affected by gender [6.50 (3.11–14.77) ng/ml in men vs. 9.172 (3.348–20.098) ng/ml in women, *p* = 0.485] or Child classification [13.19 (7.88–18.54) ng/ml in Child’s Class A, 6.62 (2.96–10.39) ng/ml in Child’s Class B, and 8.17 (3.35–20.79) ng/ml, *p* = 0.741]. Together, these findings suggest that leptin levels reflect nutritional status and insulin resistance in hospitalized cirrhotic patients.

### Serum leptin and INR are independently associated with malnutrition in hospitalized cirrhotic patients

To identify features associated with malnutrition, logistic regression was performed. Variables with *p* < 0.1 in univariate analyses were included in a backward stepwise elimination algorithm to generate the final multivariable model (Tables 3 and 4). While inflammatory cytokines and subjective measures of anorexia were not associated with malnutrition in univariate analyses, MELD score, INR, wet and dry BMI, hemoglobin and log-transformed leptin exhibited significant univariate odds ratios (*p* < 0.1). In multivariable analysis, log-transformed leptin and INR were independently associated with malnutrition, and this relationship persisted after adjusting for race, gender, and age (S4 Table). A separate analysis of interaction between INR and leptin, however, demonstrated no effect on malnutrition. AUROC measurements demonstrated fair-to-good predictive accuracy for the multivariable model (AUROC 0.797, 95% C.I. 0.674–0.920, *p* = 0.0006).

**Table 3:** Univariate logistic regression analysis for factors associated with malnutrition.

Clinical Factor	Univariate O.R. (95% CI)	*P*
**Age**	0.982 (0.934–1.032)	0.471
**Gender**	1.727 (0.565–5.283)	0.338
**Race**	0.724 (0.043–12.248)	0.823
**MELD**	1.190 (1.038–1.364)	0.012
**Sodium**	1.068 (0.929–1.227)	0.93
**Creatinine**	1.776 (0.598–5.278)	0.301
**Albumin**	1.181 (0.496–2.814)	0.38
**Bilirubin**	1.016 (0.978–1.055)	0.414
**INR**	4.836 (1.186–19.724)	0.028
**WBC count**	0.929 (0.786–1.101)	0.398
**Hemoglobin**	0.730 (0.527–1.008)	0.056
**Platelets**	0.997 (0.983–1.011)	0.632
**Wet BMI**	0.851 (0.762–0.954)	0.004
**Dry BMI**	0.258 (0.102–0.652)	0.004
**SNAQ Questionnaire**	0.994 (0.839–1.178)	0.947
**Anorexia**	1.071 (0.331–3.470)	0.908
**Walking speed**	0.944 (0.833–1.069)	0.361
**HOMA-IR**	1.014 (0.929–1.106)	0.76
**Adiponectin**	1.000 (1.000–1.000)	0.469
**Resistin**	1.000 (0.999–1.000)	0.827
**PAI-1**	0.999 (0.999–1.000)	0.334
**Amylin**	1.012 (0.990–1.033)	0.292
**Ghrelin**	1.006 (0.983–1.029)	0.604
**GIP**	1.000 (0.997–1.003)	0.824
**GLP-1**	1.001 (0.997–1.004)	0.624
**IL-6**	1.001 (0.992–1.010)	0.885
**IL-8**	1.000 (0.998–1.002)	0.691
**IL-1**	0.998 (0.988–1.008)	0.663
**TNF-α**	1.094 (0.864–1.386)	0.455
**Log-transformed leptin**	0.460 (0.255–0.829)	0.01

Univariate logistic regression analysis for factors associated with malnutrition. GIP, gastric inhibitory peptide; GLP-1, glucagon-like peptide-1; HOMA-IR, homeostatic model assessment insulin resistance; IL-1,-6,-8, interleukin-1,-6,-8; INR, international normalized ratio; MELD, Model for End-stage Liver Disease; PAI-1, plasminogen activator inhitior-1; TNF-α, tumor necrosis factor-α; WBC, white blood cell. Features with *p* < 0.1 were included in the multivariable analysis.

**Table 4:** Multivariable stepwise logistic regression analysis for factors associated with malnutrition.

Clinical Factor	Multivariate O.R. (95% CI)	*P*
**INR**	6.581 (1.330–32.569)	0.021
**Log-transformed leptin**	0.425 (0.221–0.816)	0.01

Multivariate backward stepwise logistic regression model of features associated with malnutrition. INR, international normalized ratio.

## Discussion

Malnutrition frequently develops in decompensated cirrhosis and significantly impacts survival. Yet, the underlying pathophysiologic mechanisms of malnutrition in cirrhosis are poorly understood, and clinicians lack objective biochemical tests to determine the presence and severity of malnutrition in cirrhosis. Thus, there is an urgent need to identify objective biomarkers of cirrhosis-associated malnutrition to enable early diagnosis, to monitor response to treatment, and to improve cirrhosis prognostic scores by incorporating quantitative biomarkers of malnutrition. Therefore, we evaluated biochemical, serologic, hormonal, radiographic, and anthropometric features in hospitalized cirrhotic patients to obtained insights into the pathophysiologic drivers of malnutrition in cirrhosis and to identify potential biomarkers of liver cirrhosis-associated malnutrition.

Our study provided several import insights. First, contrary to our expectation, the degree of liver disease severity in malnourished individuals was only slightly worse than in patients with malnutrition. While the two-point difference in average MELD scores between the malnourished and not malnourished group was statistically significant, it is unlikely that this small difference is clinically meaningful, since there was no difference in the prevalence of cirrhosis-related complications between the two groups. While we were unable to measure food intake, we found no difference in appetite, as determined by the SNAQ questionnaire score, between the malnourished and not malnourished groups. These findings suggest that malnourishment is not an inevitable occurrence in decompensated cirrhosis but, rather, represents a disease sub-phenotype resulting from a distinct pathophysiologic pathway.

Second, among the several cytokines and adipokines we tested, only leptin differed between patients with and without malnutrition. Leptin is a 15 kD peptide that is produced by adipocytes to regulate energy homeostasis. In healthy individuals, leptin is secreted in proportion to adipose mass and signals via hypothalamic receptors to decrease food intake and increase energy expenditure. Other regulators of leptin production include inflammatory cytokines (IL-1, TNF-α, and IL-6), glucocorticoids, and insulin [26, 27]. In our study population, malnutrition was associated with a nearly three-fold decrease in serum leptin levels despite an absence of differences in cytokine profiles, adipokine levels, gut hormone concentrations, or insulin resistance. This finding is consistent with prior studies showing correlation between serum leptin levels and body fat mass in patients with alcoholic cirrhosis [28] or cirrhosis due to viral hepatitis [29]. In addition, hypoleptinemia in malnourished cirrhotic patients was independent of liver function, as leptin levels were not correlated with MELD score. Furthermore, hypoleptinemia has been observed in other conditions associated with malnutrition including acquired and inherited lipodystrophies [30], cancer cachexia [31, 32], and cachexia due to pulmonary disease [33, 34]. As leptin is secreted by adipose tissue, hypoleptinemia likely reflects depletion of adipose tissue mass. Although malnutrition is defined as a deficiency of lean muscle, these findings suggest an important role of dysregulated fat metabolism in the pathogenesis of cirrhosis-related malnutrition.

Third, logistic regression analysis revealed that besides log-transformed leptin, INR independently associated with malnutrition in hospitalized cirrhotic patients. Malnourished patients with end-stage liver disease exhibited slightly higher MELD scores, and this was driven by differences in INR, as median bilirubin and creatinine were similar between groups. INR elevation in malnourished patients may be not only due to impaired hepatic synthetic function, but also related to altered gut microbial flora, decreased fat intake, and reduced intestinal absorption of fat-soluble Vitamin K. Given its sensitivity to nutritional factors, INR may represent a robust marker of malnutrition in chronic liver disease. Since anthropometric measures of malnutrition are difficult to interpret in the setting of ascites and edema, leptin and INR may provide clinical utility as indicators of pre-clinical malnutrition and adipose tissue reserve.

A few limitations are noted. First, there are no generally accepted definitions for malnutrition in cirrhosis. In the current study, we used readily available measures (MAMC < 23 cm and dominant hand grip strength < 30 kg) that previously exhibited 94% sensitivity and 97% negative predictive value for identifying malnutrition in cirrhotic patients [13, 14]. Using this definition, 42.3% of the current study population met criteria for malnutrition, which is comparable to reported prevalence rates in other populations. These criteria were also selected because they assess both muscle mass and function. To further validate this classification scheme, we demonstrated that malnourished patients had significant reductions in skeletal muscle index. Second, modulators of leptin secretion, including glucocorticoids and sex hormones, were not measured in the patient population; however, the effects of feeding status and diurnal variations on hormone secretion were minimized by collecting early morning samples after overnight fasting. Third, only patients with decompensated cirrhosis were studied, and the role of leptin as a biomarker of malnutrition in less advanced forms of liver disease remains unclear. Fourth, ghrelin circulates as two biologically active moieties, acyl- and des-acyl-ghrelin [35].Assessment of ghrelin levels was limited to acyl-ghrelin only, but des-acyl-grehlin has been shown to exert potentially beneficial effects against muscle loss in direct muscle injury [36]. Finally, a small sample size may have increased the probability of type II errors in hypothesis testing; despite this, we identified serum leptin and INR as robust markers of malnutrition in a well-phenotyped cohort of hospitalized cirrhotic patients.

In conclusion, low serum leptin levels and elevated INR were associated with malnutrition in hospitalized patients with decompensated cirrhosis. We propose that serum leptin should be further investigated as a potential biomarker for cirrhosis-associated malnutrition to enable early diagnosis, quantification of severity of malnutrition and monitoring response to treatment.

## Supporting Information

S1 Fig Spearman rank correlation between CT-derived and bedside anthropometric measurements. (A) Spearman rank correlation of visceral fat index with triceps skinfold thickness, dry BMI, and wet BMI. (B) Spearman rank correlation of skeletal muscle index with visceral fat index, mid-arm muscle circumference, dry BMI, and wet BMI.
(DOCX)

S2 Fig Spearman rank correlation of serum leptin with visceral fat index and skeletal muscle index. (DOCX)

S1 Table Hospital admission diagnoses of cirrhotic patients with and without malnutrition. (DOCX)

S2 Table Serum levels of metabolic factors, gut-derived hormones, cytokines and adipokines. (DOCX)

S3 Table Correlations between serum leptin levels, and nutritional, clinical, metabolic, and serologic factors. (DOCX)

S4 Table Correlations between serum leptin levels, and nutritional, clinical, metabolic, and serologic factors. (DOCX)
