# Supplementary material for: Serum Leptin Is a Biomarker of Malnutrition in Decompensated Cirrhosis
Source: PLoS One. 2016 Sep 1;11(9):e0159142. doi: 10.1371/journal.pone.0159142 (PMC5008824; doi:10.1371/journal.pone.0159142)
Supplement: S1 Fig — (A) Spearman rank correlation of visceral fat index with triceps skinfold thickness, dry BMI, and wet BMI. (B) Spearman rank correlation of skeletal muscle index with visceral fat index, mid-arm muscle circumference, dry BMI, and wet BMI. (DOCX) [file pone.0159142.s001.docx]

**
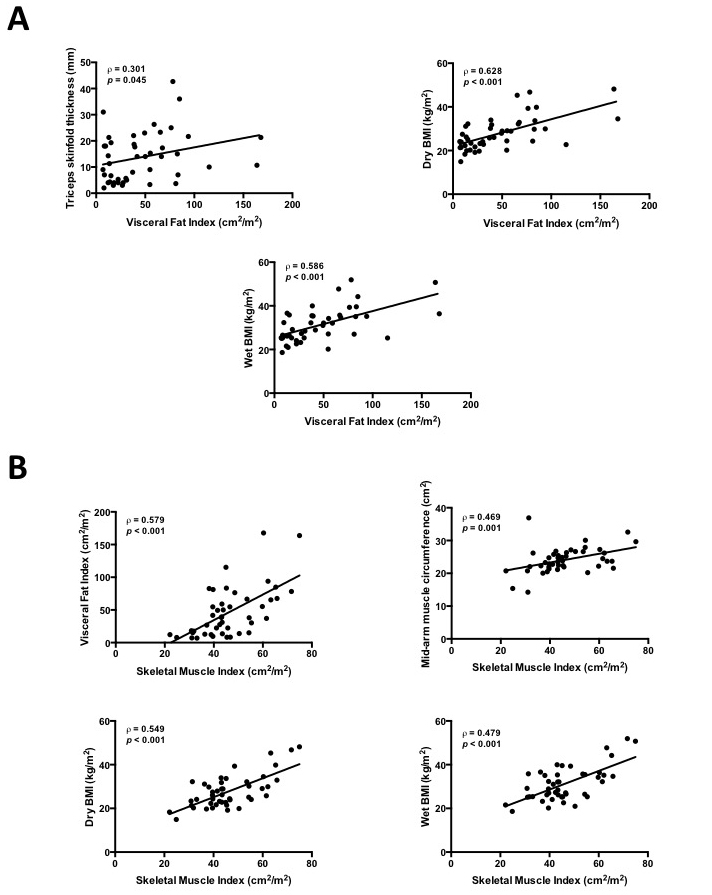
**

**S1 Figure:** (A) Spearman rank correlation of visceral fat index with triceps skinfold thickness, dry BMI, and wet BMI. (B) Spearman rank correlation of skeletal muscle index with visceral fat index, mid-arm muscle circumference, dry BMI, and wet BMI.
