# Supplementary material for: Serum Leptin Is a Biomarker of Malnutrition in Decompensated Cirrhosis
Source: PLoS One. 2016 Sep 1;11(9):e0159142. doi: 10.1371/journal.pone.0159142 (PMC5008824; doi:10.1371/journal.pone.0159142)
Supplement: S2 Fig — (DOCX) [file pone.0159142.s002.docx]

**
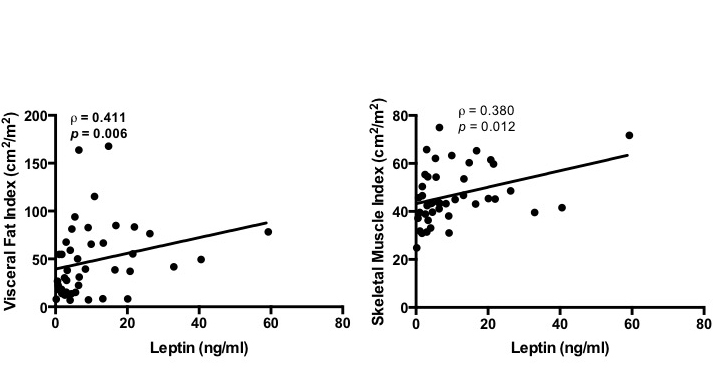
 S2 Figure:** Spearman rank correlation of serum leptin with visceral fat index and skeletal muscle index.
