# Supplementary material for: Serum Leptin Is a Biomarker of Malnutrition in Decompensated Cirrhosis
Source: PLoS One. 2016 Sep 1;11(9):e0159142. doi: 10.1371/journal.pone.0159142 (PMC5008824; doi:10.1371/journal.pone.0159142)
Supplement: S1 Table — (DOCX) [file pone.0159142.s003.docx]

**S1 Table: Hospital admission diagnoses of cirrhotic patients with and without malnutrition**

| Hospital Admission Diagnoses | Total  (N=52) | Malnourished  (N=22) | No malnutrition  (N=30) | *P* |
| --- | --- | --- | --- | --- |
| Gastrointestinal Bleeding, n (%)  Hepatic encephalopathy, n (%)  *Volume derangements, n (%)*  Ascites or Hepatic Hydrothorax  Acute kidney injury  *Infections, n (%)*  Spontaneous bacterial peritonitis/ empyema  Respiratory tract infections  Urinary tract infections  Bacteremia/Endocarditis  Enteritis or colitis | 3 (5.8%)  23 (44.2%)  40 (76.9%)  20 (38.5%)  8 (15.4%)  3 (5.8%)  3 (5.8%)  4 (7.7%)  6 (11.5%)  3 (5.8%) | 0 (0%)  11 (50.0%)  18 (81.8%)  10 (45.5%)  2 (9.1%)  1 (4.5%)  2 (9.1%)  2 (9.1%)  2 (9.1%)  1 (4.5%) | 3 (10%)  12 (40.0%)  22 (73.3%)  10 (33.3%)  6 (30.0%)  2 (6.7%)  1 (3.3%)  2 (6.7%)  4 (13.3%)  2 (6.7%) | 0.253  0.576  0.526  0.403  0.422  1.000  0.567  1.000  0.494  1.000 |

**Supplementary Table S1:** Hospital admission diagnoses of cirrhotic subjects with and without malnutrition.
