# Supplementary material for: Serum Leptin Is a Biomarker of Malnutrition in Decompensated Cirrhosis
Source: PLoS One. 2016 Sep 1;11(9):e0159142. doi: 10.1371/journal.pone.0159142 (PMC5008824; doi:10.1371/journal.pone.0159142)
Supplement: S2 Table — (DOCX) [file pone.0159142.s004.docx]

**S2 Table: Serum levels of metabolic factors, gut-derived hormones, cytokines and adipokines**

| Characteristics | Total  (N=52) | Malnourished  (N=22) | No malnutrition  (N=30) | *P* |
| --- | --- | --- | --- | --- |
| Diabetes (n, %)  Glucose (mg/dl)  Insulin (µU/ml)  HOMA-IR  Amylin (pg/ml)  Ghrelin (pg/ml)  GIP (pg/ml)  GLP-1 (pg/ml)  IL-1β (pg/ml)  IL-6 (pg/ml)  IL-8 (pg/ml)  TNF-α (pg/ml)  GM-CSF (pg/ml)  Adiponectin (µg/ml)  Leptin (ng/ml)  PAI-1 (ng/ml)  Resistin (ng/ml) | 25 (48.1%)  119 (98-153)  24.69 (11.07-36.12)  6.94 (3.17-12.68)  41.59 (18.41-58.30)  15.21 (6.24-35.27)  110.01 (52.05-232.75)  390.18 (292.06-481.36)  0.45 (0.45-5.64)  41.05 (22.87-98.38)  51.19 (30.53-105.82)  0.17 (0.17-1.35)  0.34 (0.29-0.54)  27.62 (18.74-43.59)  7.39 (3.15-16.76)  63.15 (48.18-90.69)  54.14 (37.24-67.44) | 10 (45.5%)  114 (88-145)  26.98 (12.06-32.11)  7.01 (3.37-11.91)  42.12 (18.44-62.42)  19.06 (9.13-29.13)  133.95 (53.70-251.60)  402.41 (331.91-485.42)  0.45 (0.45-2.39)  43.51 (27.50-106.67)  90.22 (40.30-133.77)  0.17 (0.17-0.17)  0.34 (0.29-0.39)  26.92 (19.93-60.01)  3.88 (1.52-11.02)  60.77 (51.29-83.07)  55.53 (39.40-81.01) | 15 (50.0%)  123 (104-164)  22.65 (10.67-37.37)  6.94 (3.03-12.75)  39.91 (18.88-51.93)  13.82 (4.62-28.26)  92.25 (52.63-197.47)  351.34 (278.95-473.64)  0.45 (0.45-11.33)  40.81 (20.68-81.92)  47.41 (26.55-74.15)  0.17 (0.17-1.39)  0.39 (0.29-0.55)  28.79 (18.15-43.49)  10.39 (5.46-21.06)  64.64 (46.51-91.95)  51.48 (37.24-66.34) | 0.483  0.182  0.984  0.937  0.488  0.298  0.649  0.342  0.553  0.440 0.122  0.288  0.503  0.759  0.011  0.553  0.621 |

**Supplementary Table S2:** Circulating levels of metabolic factors, gut-derived hormones, cytokines, and adipokines. GIP, gastric inhibitory peptide; GLP-1, glucagon-like peptide-1; GM-CSF, granulocyte-macrophage colony stimulating factor; HOMA-IR, homeostatic model assessment insulin resistance; IL-1,-6,-8, interleukin-1,-6,-8; PAI-1, plasminogen activator inhitior-1; TNF-α, tumor necrosis factor-α.
