# Supplementary material for: Serum Leptin Is a Biomarker of Malnutrition in Decompensated Cirrhosis
Source: PLoS One. 2016 Sep 1;11(9):e0159142. doi: 10.1371/journal.pone.0159142 (PMC5008824; doi:10.1371/journal.pone.0159142)
Supplement: S3 Table — (DOCX) [file pone.0159142.s005.docx]

**S3 Table: Correlations between serum leptin levels, and nutritional, clinical, metabolic, and serologic factors**

| Characteristics | Spearman correlation coefficient | *P* |
| --- | --- | --- |
| Wet weight  Dry weight  Wet BMI  Dry BMI  Mid-arm circumference  Triceps skin fold thickness  Mid-arm muscle circumference  Hand grip strength  SNAQ Questionnaire  15 foot walking time  MELD  Sodium  BUN  Creatinine  Albumin  Bilirubin  INR  WBC  Platelets  Glucose  Insulin  HOMA-IR  Adiponectin  Resistin  PAI-1  Amylin  Ghrelin  GLP-1  GIP  IL-6  IL-8  IL-1  TNF-α  GM-CSF | 0.361  0.367  0.544  0.526  0.458  0.556  0.321  -0.006  -0.071  0.233  -0.212  0.076  0.018  -0.090  0.220  -0.166  -0.0003  -0.120  -0.069  0.117  0.415  0.356  -0.174  -0.182  -0.093  0.208  -0.138  0.096  -0.061  -0.228  -0.147  -0.134  -0.088  -0.047 | 0.010  0.009  < 0.001  < 0.001  0.001  < 0.001  0.023  0.968  0.626  0.103  0.139  0.601  0.902  0.536  0.129  0.250  0.998  0.199  0.636  0.419  0.003  0.011  0.227  0.206  0.522  0.148  0.340  0.509  0.670  0.112  0.310  0.355  0.543  0.745 |

**Supplementary Table S3:** Spearman rank correlation of serum leptin with patient characteristics, nutritional factors, metabolic hormones, and inflammatory factors.
