# Supplementary material for: Serum Leptin Is a Biomarker of Malnutrition in Decompensated Cirrhosis
Source: PLoS One. 2016 Sep 1;11(9):e0159142. doi: 10.1371/journal.pone.0159142 (PMC5008824; doi:10.1371/journal.pone.0159142)
Supplement: S4 Table — (DOCX) [file pone.0159142.s006.docx]

**S4 Table: Multivariable stepwise logistic regression analysis for factors associated with malnutrition after correction for age and gender**

| Clinical Factor | Univariate O.R. (95% CI) | *P* |
| --- | --- | --- |
| INR  Log-transformed leptin  Age  Gender | 6.412 (1.263 – 32.559)  0.399 (0.193 – 0.826)  1.001 (0.946 – 1.076)  2.248 (0.571 – 8.847) | 0.025  0.013  0.789  0.247 |

**Supplementary Table S4:** Multivariable backward stepwise logistic regression model of features associated with malnutrition after adjustment for age and gender.
